# Homocysteine: Biochemistry, Molecular Biology, and Role in Disease 2021

**DOI:** 10.3390/biom13071111

**Published:** 2023-07-13

**Authors:** Guzel Sitdikova, Anton Hermann

**Affiliations:** 1 Department of Human and Animal Physiology, Institute of Fundamental Medicine and Biology, Kazan Federal University, 420008 Kazan, Russia; 2 Department of Biosciences, University Salzburg, Hellbrunnerstr 34, A-5020 Salzburg, Austria; anton.hermann@plus.ac.at

Homocysteine is increasingly recognized as an important molecule in a wide variety of cellular functions. Homocysteine levels can be altered in different ways, including enzyme disorder cofactor deficiency; excessive methionine intake; specific diseases, such as chronic renal failure, hypothyroidism, and anemia; and intake of specific drugs, such as cholestyramine, methotrexate, oral contraceptive pills, phenytoin, carbamazepine, or metformin. Clinical studies indicate an association between high homocysteine levels and different pathological conditions, such as endothelial dysfunctions, myocardial infarction, ischemic injury, age-dependent diseases (i.e., dementia), inflammation or migraine. The increased levels of plasma homocysteine, called hyperhomocysteinemia (hHcy), are classified as mild (15–30 μmol/L), medium (30–100 μmol/L) or severe (more than 100 μmol/L). However, the causal relationship between several disorders and hHcy remains unclear in many cases. The level of the gasotransmitter hydrogen sulfide (H_2_S) decreases under hHcy conditions, which may mediate homocysteine-induced neurotoxicity; on the other hand, an increase in H_2_S may play a neuroprotective role.

In this second SI devoted to homocysteine, we focused on the molecular mechanisms of homocysteine pathophysiology, reliability of animal models of hHcy, possibility of using vitamin B in the prevention of hHcy and its consequences, and even proposed homocysteine as an initial amino acid which derivative-homocysteine thiolactone allowed the production of dipeptides in the evolution of life.

This Special Issue includes three original papers and three reviews comprising forty authors who are experts in the field.

In the paper of Youssef-Saliba et al., homocysteine was suggested to be involved in protein synthesis in the early evolution of life on Earth. Despite the fact that homocysteine today is not a proteinogenic amino acid, it is still important in the metabolism of the proteinogenic amino acids cysteine and methionine. When it is mistakenly introduced into the process of synthesizing a peptide in the ribosome, it is rejected by an editing mechanism that produces thiolactone, which, as homocysteine thiolactone, may play role in protein damage. In this work, the cyclization of homocysteine into thiolactone in water was shown experimentally and supported by theoretical considerations. Moreover, homocysteine thiolactone could react with amino acids to produce dipeptides, which are proposed as an interesting molecular building block at the origin of life on Earth [1].

In the study of Prtina et al., the effects of high doses of vitamin D supplementation on the levels of homocysteine, vitamin B_12_, and folate, pro-inflammatory and anti-inflammatory cytokines in the sera of patients with plaque psoriasis were studied. Besides the local inflammatory changes in the skin, psoriatic patients show symptoms of systemic inflammation frequently characterized by hHcy. In addition, an inverse relationship between vitamin D and homocysteine was shown as vitamin D directly affects the transcription of genes involved in homocysteine metabolism. Three months of vitamin D supplementation significantly increased serum vitamin B_12_ concentrations, but serum homocysteine and folate values were significantly lower compared to the baseline. At the same time, skin lesions were clinically improved, along with a decrease in the production of pro-inflammatory cytokines and an increase in the production of anti-inflammatory cytokines [2].

Cardiovascular diseases (CVDs), particularly ischemic heart and brain diseases, are the leading cause of death and the main cause of disability today. In two reviews, the role of homocysteine in myocardial infarction—heart failure and cerebral ischemic injury—was analyzed [3,4].

Bajic et al., 2022, summarized evidence of homocysteine as a marker of CVD and a risk factor, in particular, for stroke, myocardial infarction, heart failure, cancer, Alzheimer’s disease, and atherosclerosis. An elevated homocysteine concentration induces endothelial dysfunctions, damaging the blood vessel wall, which results in a change in its properties from anticoagulant to a procoagulant state. Moreover, homocysteine activates coagulation factors and impairs the balance between vasodilators and vasoconstrictor. Homocysteine induces toxic effects due to the inhibition of Na^+^-,K^+^-ATPase, lowering levels of gasotransmitters (NO, CO, H_2_S), the overstimulation of n-methyl-D-aspartate (NMDA) receptors, inducing inflammation and oxidative stress, and mitochondrial dysfunction in cardiac tissue. The supplementation of vitamin B6 and folic acid could lower homocysteine levels and improve heart function in experimental models of myocardial infarction and heart failure respiration [3].

^1^H nuclear magnetic resonance (NMR) metabolomics is a fast-developing approach which allows for the identification and quantification of metabolites in different tissues and blood plasma. In the review by Baranovicova et al., 2022, the authors characterized their own work and data from the literature regarding the metabolic changes induced by cerebral ischemic injury in rodents, which include the decrease in the neurotransmitter pool with an elevation of the glutamine level. In remote organs, such as the heart and liver, and in the blood plasma, post-ischemically induced hyperglycemia with a ketosis-like state was observed. In rats with hHcy, cerebral ischemia induced more pronounced alterations in the metabolomic state manifested by the apparent decline in the levels of many amino acids. In addition, ischemic attack aggravates hHcy-induced neurodegenerative processes observed by histological patterns and eventually leads to the manifestation of the development of Alzheimer’s disease-like neuropathology [4]. 

Nieraad et al., 2021, presented a review where the authors discussed in detail the reactions of the C1 metabolism, kinetics of enzymes, and made a conclusion about four frequent cases of C1 metabolic disorders, including deficiency of B-group vitamins, polymorphism of MTHFR or CBS in heterozygous form, lifestyle factors, and oxidative stress. In the second part, the authors critically reviewed the animal models of hHcy, which allowed for consideration of the preclinical evidence on the impact of hHCY on cognitive performance and decline. Despite controversial findings, especially in clinical studies, preclinical studies indicate a causal link between hHcy and cognition-related—especially dementia-like—disorders. Therefore, it is necessary to organize large-scale, well-designed clinical studies in order to elucidate if the normalization of homocysteine levels may serve as a preventive or therapeutic approach in human pathologies [5].

Finally, the original research of Gerasimova et al., 2022, aimed to reveal the role of homocysteine in the central and peripheral mechanisms of migraine. Behavioral correlates of headache and spreading cortical depolarization (CSD) in a migraine model induced by the administration of the nitric oxide (NO) donor nitroglycerin were studied in rats with prenatal hHcy. Animals with hHcy were characterized by mechanical hyperalgesia, high-level anxiety, and photophobia, indicating central sensitization. Higher levels of neuronal activity in the somatosensory cortex along with a lower threshold of CSD generation indicated cortical hyperexcitability. In addition, rats with hHcy were more sensitive to the acute or chronic intermittent administration of nitroglycerine. These data support clinical data, indicating a causal link between homocysteine levels and higher risk of migraine headaches [6].
